# Response of Bud Banks of a Freshwater Herbaceous Marsh Plant (*Glyceria Spiculosa*) to Heterogeneous Habitat: Field Observations and Experiment

**DOI:** 10.1002/ece3.71522

**Published:** 2025-06-17

**Authors:** Mengdie Zhou, Changwei Zhang, Xianglong Jin, Qiyao Zhang, Peng Zhang, Anni Bao, Yanjing Lou

**Affiliations:** ^1^ Jilin Provincial Joint Key Laboratory of Changbai Mountain Wetland and Ecology Northeast Institute of Geography and Agroecology, Chinese Academy of Sciences Changchun China; ^2^ No. 3 Middle School of Yichang Yichang China; ^3^ University of Chinese Academy of Sciences Beijing China; ^4^ College of Geographic Sciences Changchun Normal University Changchun China

**Keywords:** bud banks, clonal plants, environmental changes, flooding condition, nitrogen addition

## Abstract

Exploring plant adaptation strategies under habitat variations from the perspective of bud banks is essential for understanding vegetation regeneration under global changes. However, the response of wetland plant bud banks to combined water and nutrient fluctuations remains unclear. In this study, field surveys across three vegetation zones (wet meadow, tussock marsh, and marsh) and pot experiments with three flooding periods (no flooding, flooding for 1–5 weeks, and flooding for 6–10 weeks), four nitrogen levels (1, 10, 30, and 100 mg/L) and two nitrogen forms (ammonium and nitrate) of *Glyceria spiculosa* bud banks were conducted. Our results showed that rhizome internode buds (accounting for 91%) dominated the bud bank compared with rhizome apical and tiller buds in natural habitats, and bud density in marsh (2475.64 No./m^2^) was higher than that in tussock marsh (1225.33 No./m^2^) and wet meadow (1583.43 No./m^2^). In the pot experiment, the proportion of tiller buds exceeds that of rhizome internode buds. The densities of total buds and tiller buds increased with increasing nitrogen levels, and the effect of ammonium nitrogen is higher than that of nitrate nitrogen. Overall, the impact of nitrogen is greater than that of flooding. These findings reveal that adjusting bud bank composition and density is a core adaptive strategy of wetland clonal plants to adapt to changing environmental conditions. Meanwhile, management and regulation of nitrogen fertilizers (including nitrogen form and level) and flooding periods should be paid more attention to in wetland restoration to maintain bud bank‐mediated regeneration capacity of plant populations.

## Graphical abstract

We illustrate the adaptation strategies of *Glyceria spiculosa* to water and nitrogen variations from a new perspective of bud banks. These findings reveal that adjusting bud bank composition, that is, prioritizing rhizome buds in flooding habitats and tiller buds under nitrogen enrichment, is a core adaptive strategy for clonal plants. The dominance of ammonium nitrogen in promoting bud proliferation underscores nutrient form as a critical driver of wetland resilience, offering novel insights for managing ecosystems under intensified nitrogen deposition and hydrological shifts. Specifically, conservation strategies should account for nitrogen form preferences in wetland restoration, while hydrological management must consider seasonal flooding patterns to maintain bud bank‐mediated regeneration capacity.

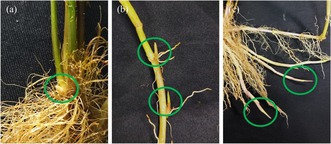

## 1 Introduction

The bud banks consist of all buds which may be used for vegetative regeneration (Klimešová and Klimeš 2007), playing a pivotal role in plant population regeneration and community dynamics, especially when following disturbances and environmental change (Ferraro et al. 2022). Bud formation is complex and depends not only on the genetic characteristics of the parent plant, but is also closely linked to habitat conditions (Ott et al. 2019). To adapt to variable environments, plants may adjust bud demography to ensure the successful completion of their life cycles (Deng et al. 2013). For example, the proportion of tiller buds and rhizome buds changes under different habitat conditions (Yang et al. 2022). So, the dynamics of bud banks directly influence plant population renewal, help predict vegetation succession under varying environments, and serve as a crucial indicator for assessing ecosystem recovery potential.

In wetland ecosystems where clonal plants predominate, the bud bank has emerged as the primary reproductive form for wetland plants (Sosnová et al. 2010). However, climate change and intensified agricultural activities have collectively increased the frequency of droughts and flooding in wetlands. Meanwhile, intensified atmospheric nitrogen deposition and widespread nitrogen fertilizer use have exacerbated nitrogen enrichment in these ecosystems. These combined pressures (droughts/flooding and nitrogen enrichment) now constitute the primary stress factors for wetland ecosystems (Zhou et al. 2023; Raza et al. 2020). Regrettably, the response of wetland plant bud banks to these habitat alterations remains understudied and warrants urgent attention.

The effect of hydrological conditions on bud bank characteristics in freshwater wetlands has been demonstrated by many studies (Ding et al. 2019; Chen et al. 2015). For example, frequent flooding disturbances promote the formation of a larger bud bank for *Phalaris arundinacea* to facilitate rapid regeneration (Chen et al. 2022), and drought conditions lead to a reduction in bud bank density (Luo et al. 2023; Qian et al. 2022; Dalgleish and Hartnett 2006). Rhizome buds proliferate under high water conditions and exhibit great sensitivity to variations in water levels, whereas tiller buds are more common under low water conditions (Wang et al. 2008). Moreover, the impact of hydrological conditions on bud bank formation can vary depending on the stage of plant growth. For instance, early‐season flooding has been shown to significantly inhibit the clonal reproduction of *Carex lasiocarpa* (Wang et al. 2009), while flooding in the late growing season prompts plants to allocate more resources to vegetative reproduction (Li et al. 2020). Given these findings, understanding how plants adjust their bud bank dynamics in response to changing hydrological conditions is crucial for predicting population dynamics and community patterns of wetland plants.

The bud bank dynamic of clonal plants is also affected by nutrient conditions (Zheng et al. 2018; Ott and Hartnett 2012). Generally speaking, higher nitrogen concentrations tend to enhance the density of bud banks (Dalgleish and Hartnett 2008; Dalgleish et al. 2008), while the bud number in plants tends to reduce under environments with low nitrogen (Ye et al. 2006). Moreover, different types of buds respond differently to nitrogen conditions. Yu et al. (2022) discovered that high nitrogen levels promote tiller bud production but inhibit rhizome and bulb bud formation. Meanwhile, differences in nitrogen forms may lead to distinct bud bank dynamics. Compared to nitrate assimilation, ammonium assimilation requires less energy and can accelerate cell division, thereby exerting a stronger promoting effect on root and plant growth (Bloom et al. 2003). However, whether ammonium nitrogen can also more effectively promote bud bank formation remains underexplored.

*Glyceria spiculosa* is a perennial herbaceous plant that grows in freshwater marshes and is widely distributed in China, East Russia, and North Korea (Bai et al. 2021). Field investigations in Northeast China show that this species occupies a wide niche across different water levels and is distributed in wet meadows, tussock marshes, and marshes along flooding gradients (Lou et al. 2018). Due to its wide water depth amplitude, *G. spiculosa* is widely used as an ideal experimental material for studying wetland plant responses to environmental changes (Tian et al. 2022; Bai et al. 2021; Luo et al. 2010). In this study, we conducted a field survey along natural vegetation zones gradient and designed a pot experiment simulating flooding periods and nitrogen addition to investigate how bud banks of wetland plant *G. spiculosa* respond to environmental changes. Specifically, the objectives were to: (1) identify variations in bud bank types and densities across different vegetation zones; (2) examine the bud bank characteristics under simulated flooding and nitrogen addition; and (3) determine the key environmental factors driving variations in bud banks in the field and experiment.

## 2 Materials and Methods

### 2.1 Study Area

The field investigation was conducted at the Sanjiang Marsh Wetland Ecosystem National Field Scientific Observation and Research Station (Sanjiang Station, 47°36′ N, 133°30′ E) and the Honghe National Nature Reserve (Honghe Reserve, 47°47′ N, 133°37′ E) in Heilongjiang Province, China (Figure S1). These two sites have typical vegetation zones (from wet meadows to tussock marshes and then to marshes along increasing water levels gradient) of the Sanjiang Plain marsh (Lou et al. 2015). Within these zones, wet meadows dominated by *Deyeuxia angustifolia* occupy the highest elevations, marshes dominated by *Carex lasiocarpa* and *Carex pseudocuraica* are situated at the lowest elevation, and tussock marshes dominated by *Carex meyeriana* and *Carex appendiculata* are in the middle.

### 2.2 Field Sampling

Field sampling was carried out in late August and early September 2023. A total of 11 sample sites were selected (5 at Sanjiang Station and 6 at the Honghe Reserve). In each site, samplings were designed along vegetation zones from wet meadow to tussock marsh and then to marsh. Within each zone, a quadrat dominated by *G. spiculosa* was selected and substrate cubes (0.5 m × 0.5 m × 0.3 m) were excavated from the subsurface. The latitude, longitude, and water depth of each quadrat were precisely documented. Meanwhile, surface water and soil samples were collected and then promptly transported to the laboratory at Sanjiang Station. The soil samples were air‐dried and the water samples were kept frozen for subsequent processing and analysis.

### 2.3 Pot Experimental Design

In the early growing season (May), ramets of *G. spiculosa* were collected in Sanjiang Station and transplanted to the Northeast Institute of Geography and Agroecology, Chinese Academy of Sciences (44°00′ N, 125°23′ E). Before the experiment, healthy ramets with relatively uniform size (initial plant height of 33.21 ± 2.94 cm) were selected and planted in plastic buckets (38 cm in height and 35 cm in diameter) filled with 20 cm of prewashed river sand, with one ramet per bucket. The transplanted ramets were then randomly positioned in a greenhouse (temperature: 25.04°C ± 3.13°C; humidity: approximately 40%–50%). To exclude damaged ramets, the pots were placed in the greenhouse for 1 week before the start of the experiment.

Three flooding periods (no flooding throughout, flooding in weeks 1–5, flooding in weeks 6–10), four nitrogen levels (1, 10, 30, and 100 mg/L), and two nitrogen forms (ammonium and nitrate) were set up based on reference to flooding conditions and nitrogen levels in field conditions and relevant literature (Yang et al. 2018; Yu et al. 2015). Five replicates for each treatment, a total of 120 samples from 24 treatments (Figure S2). The control water level in the flooding treatments was set at 0 cm and the simulated flooding depth was 10 cm above the substrate surface. Ammonium sulfate ((NH_4_)_2_SO_4_) and calcium nitrate tetrahydrate (Ca(NO_3_)_2_·4H_2_O) were used to simulate ammonium and nitrate nitrogen in nitrogen form treatments, respectively. Nitrogen solutions were prepared at concentrations of 1, 10, 30, and 100 mg/L. The absolute amounts of (NH_4_)_2_SO_4_ and Ca(NO_3_)_2_·4H_2_O added per liter of solution for each nitrogen concentration are detailed in Table S1. To compensate for evaporation, water was added daily to maintain a fixed water level. Additionally, a modified Hoagland's nutrient solution (Table S2) with different nitrogen concentrations was replaced weekly in each bucket to meet the experimental treatments' requirements (Bai et al. 2021). The experiment lasted for 12 weeks, with no flooding in weeks 11–12, only maintaining substrate in a moist state. At the end of the experiment, plants were carefully excavated from the substrate, rinsed clean with water, packed into plastic bags, and then brought back to the laboratory for counting the number of buds in each bucket.

### 2.4 Measurements

**FIGURE 1:**
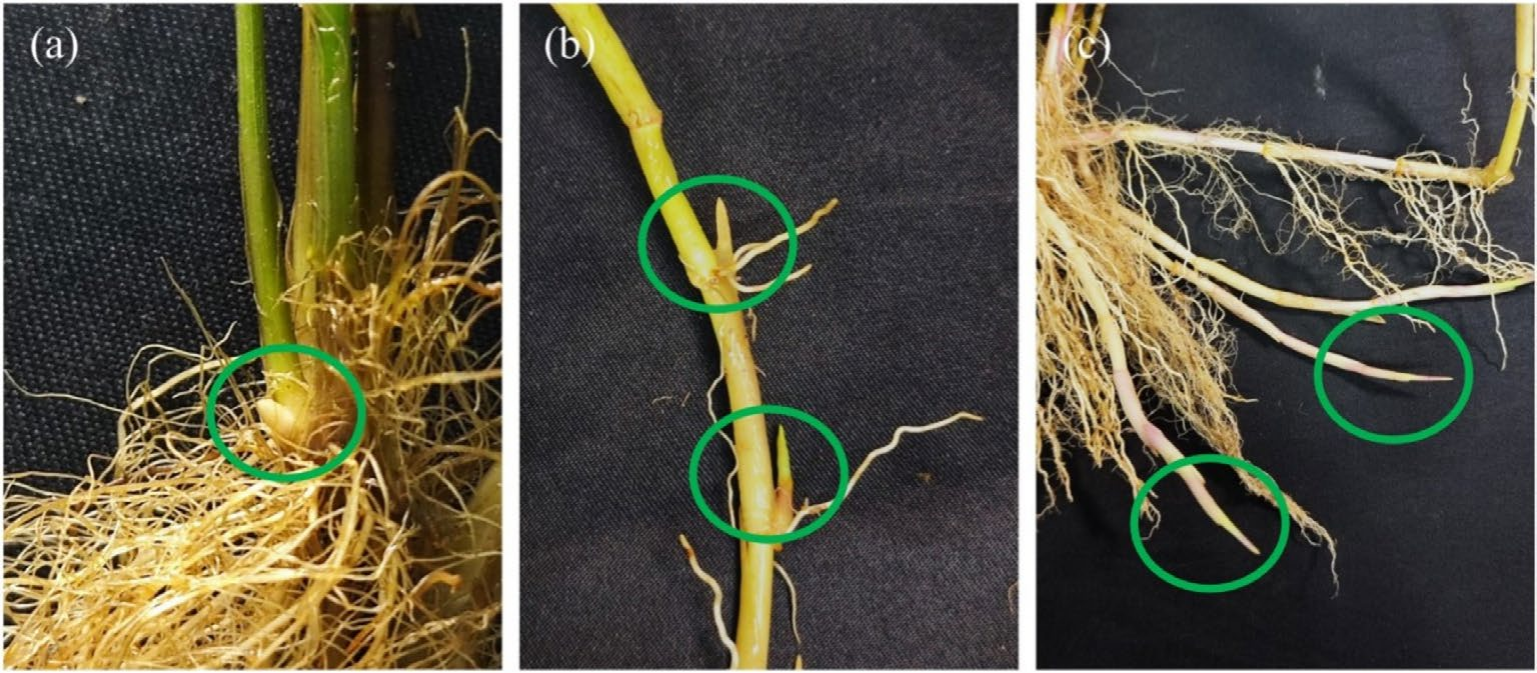
(a) Tiller buds, (b) rhizome internode buds and (c) rhizome apical buds of *G. spiculosa*.

In the laboratory, we cleaned the collected substrate cubeagain, such as removing the dead skin from the surface of the rhizomes, in order to clearly locate the buds. Subsequently, based on the location of the buds, we categorized these buds into tiller buds (buds growing vertically upward from the base of the plant), rhizome internode buds (buds located on the rhizome internodes), and rhizome apical buds (buds located at the tip of the rhizome) (Figure 1). To ensure the accuracy and detailedness of the data, we recorded the number of *G. spiculosa* buds one by one under the naked eye according to the bud type.

The soil samples were air‐dried, ground, and sieved through a 100‐mesh sieve (with a pore size of approximately 150 μm). Then digestion was performed using the hydrogen peroxide‐sulfuric acid method (H_2_O_2_‐H_2_SO_4_ digestion) to determine total nitrogen and total phosphorus; extraction was carried out with a 2 mol/L KCl solution to determine ammonium and nitrate nitrogen (The State Forestry Administration 2015). All digested samples were then analyzed using a continuous flow analyzer (SAN++, SKALAR, Netherlands). For water samples, the potassium persulfate oxidation‐ultraviolet spectrophotometric method was used to determine total nitrogen, the potassium persulfate digestion method for total phosphorus, the Nessler's reagent spectrophotometric method for ammonium nitrogen, and the ultraviolet spectrophotometry for nitrate nitrogen (The State Environmental Protection Administration 2002). All of the colorimetric analyses of the four water quality parameters were performed by UV–visible spectrophotometer (UV‐2700i, SHIMADZU, Japan).

### 2.5 Statistical Analyses

For the field survey data, a mixed‐effects model was employed to assess the impacts of natural vegetation zones and habitat factors on the bud bank density of *G. spiculosa* in natural habitats. In this process, natural vegetation zones and habitat factors were set as fixed factors, while the sample plots served as random factors. The corresponding model was constructed using the “lme4” package (Bates et al. 2015). Subsequently, the “effectsize” package was utilized to standardize the regression coefficients (Ben‐Shachar et al. 2020), the “lmerTest” package was applied to obtain *p*‐values (Kuznetsova et al. 2017), and the anova function was used for parameter significance testing. Then, the “emmeans” package was employed for Tukey's post hoc test (Lenth 2023). Correlation matrices were visualized using heatmaps, where color intensity and direction reflected correlation strength. For the pot experiment data, taking the initial plant height as a covariate, covariance analysis was conducted to explore the effects of flooding period (FP), nitrogen form (NF), nitrogen level (NL), and their interactions on the bud bank density of *G. spiculosa*. Tukey's post hoc test was then performed. Additionally, to evaluate the relative importance of water and nitrogen variables on the bud bank, the “relaimpo” package was employed to calculate the relative effect of nitrogen level, nitrogen form, and flooding period on bud bank density (Grömping 2006). All the above analyses were conducted in R 4.3.0 (R Core Team 2023).

## 3 Results

### 3.1 Variations in Bud Bank Along Natural Vegetation Zones

**FIGURE 2:**
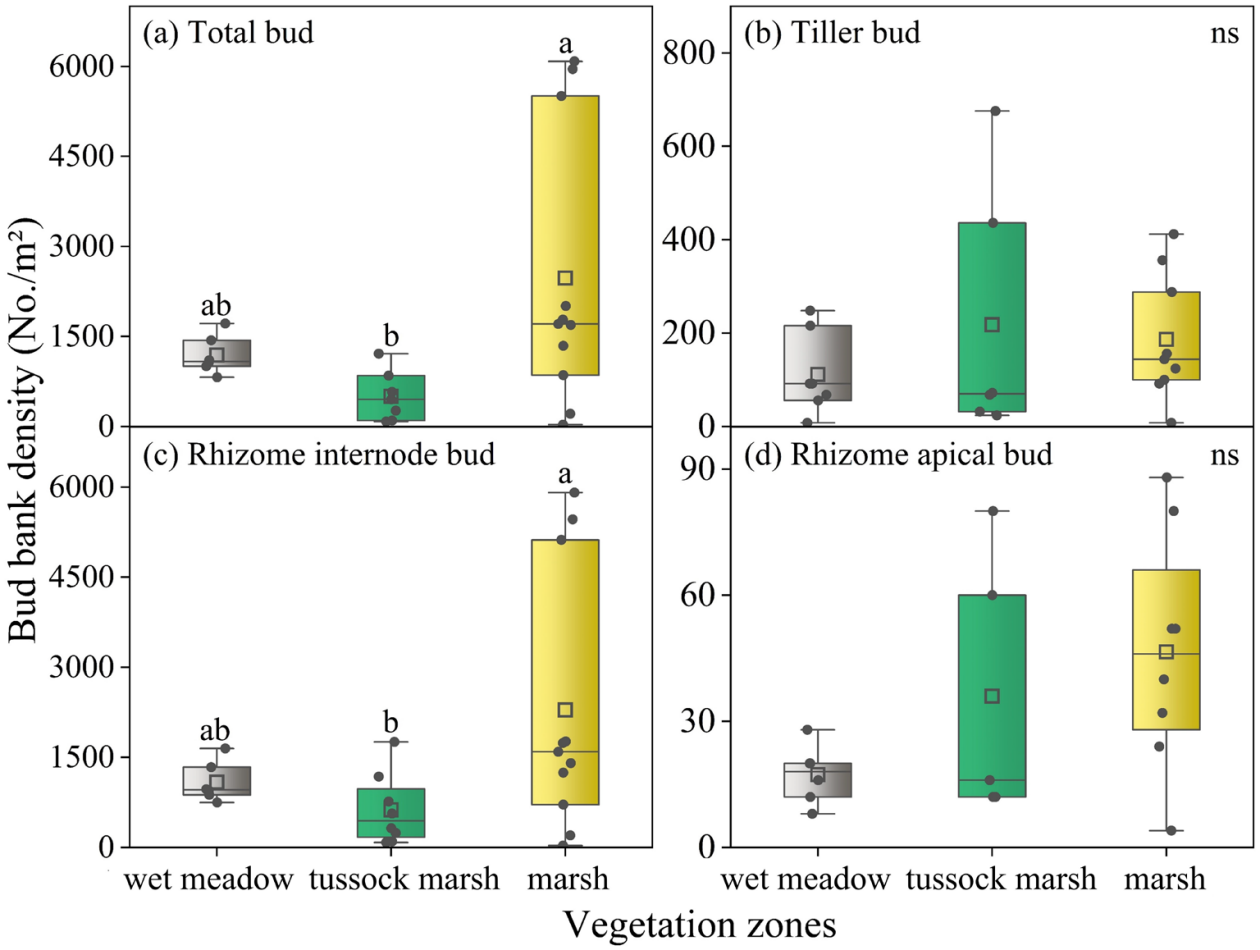
Variation in bud bank density on a gradient of wetland vegetation zones. Different lowercase letters indicate significant (*p* < 0.05) differences between vegetation zones; ns: Not significant (*p* > 0.05). The box shows the interquartile range (IQR) with the median line; whiskers extend to the most extreme data points within 1.5 × IQR of the box. The square symbol within the box indicates the mean. Error bars represent ± SE.

In natural habitats, the total bud density averaged 1827.56 No./m^2^. Rhizome internode bud constituted the primary component of the bud bank (accounting for 91.03%, 1663.7 No./m^2^), while tiller bud and rhizome apical bud represent 7.64% (139.56 No./m^2^) and 1.33% (24.30 No./m^2^) of the total bud bank, respectively. Significant differences in total bud density and rhizome internode bud density were observed among natural wetland vegetation zones (Table 1, *p* < 0.05). The marsh zone had significantly higher bud densities (total: 2475.64 No./m^2^, rhizome internode: 2289.09 No./m^2^) than the tussock marsh (total: 1225.33 No./m^2^, rhizome internode: 1060.00 No./m^2^), with the wet meadow zone (total: 1583.43 No./m^2^, rhizome internode: 1457.14 No./m^2^) in the middle (Figure 2).

**TABLE 1:** Results of ANOVA analysis of vegetation zones and bud bank density.

Bud bank density	df	*F*	*p*
Total bud	2	5.292	**0.020**
Tiller bud	2	0.356	0.708
Rhizome internode bud	2	4.865	**0.025**
Rhizome apical bud	2	2.553	0.123

*Note:* The bolded value represents *p* < 0.05.

### 3.2 Variations in Bud Bank Under Flooding and Nitrogen Treatments

Under the simulated flooding period and nitrogen addition treatments, the average density of the total bud of *G. spiculosa* was 11.88 No./plant, with a maximum of 52 No./plant. Among these bud types, tiller buds accounted for 58.72% (6.98 No./plant) of the total bud bank, followed by rhizome internode buds and rhizome apical buds (accounting for 32.33% and 8.95% of the total bud bank, respectively).

**FIGURE 3:**
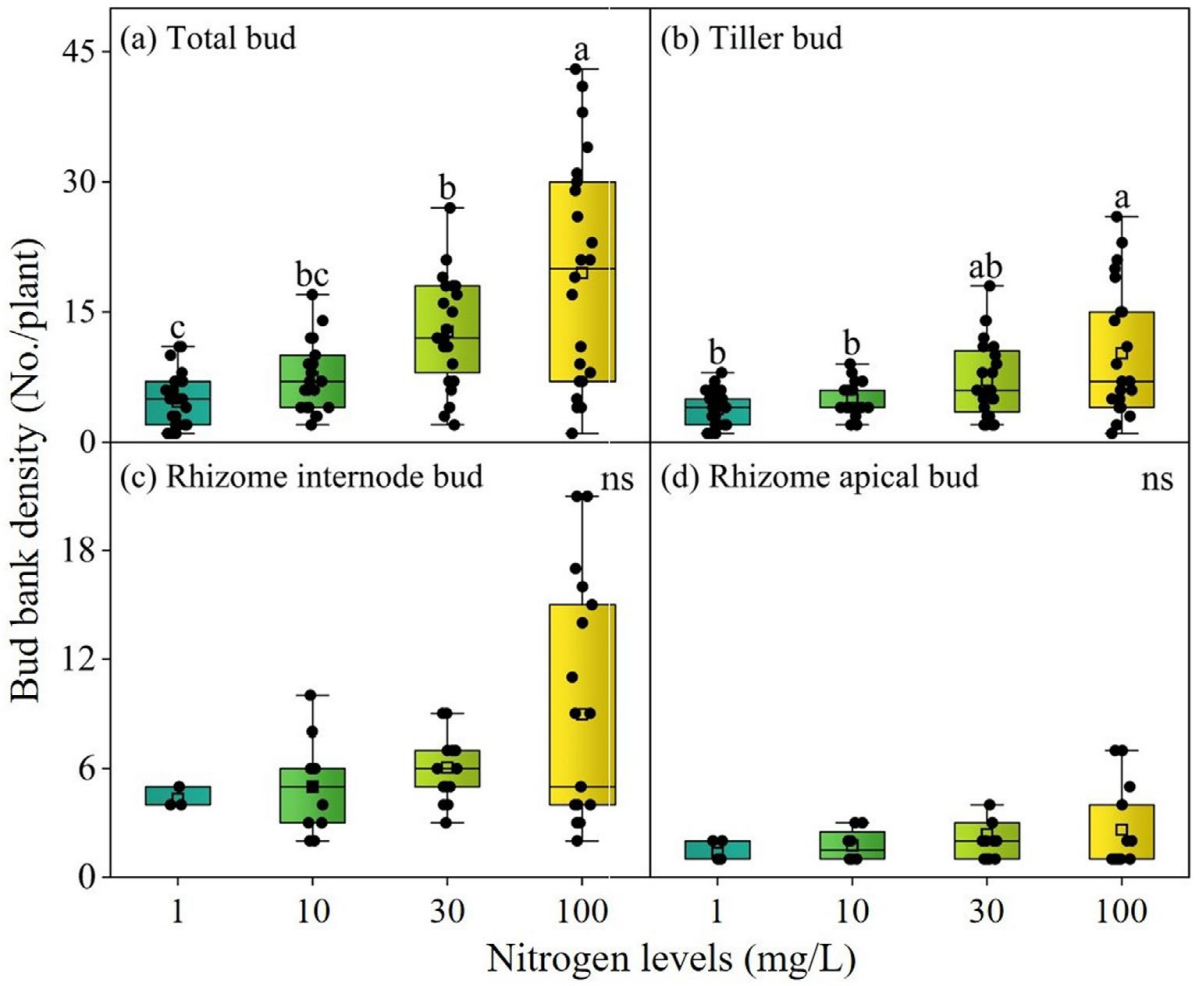
Variation in bud bank density under different nitrogen level treatments. Different lowercase letters indicate significant (*p* < 0.05) differences between different nitrogen levels; ns: Not significant (*p* > 0.05). The box shows the interquartile range (IQR) with the median line; whiskers extend to the most extreme data points within 1.5 × IQR of the box. The square symbol within the box indicates the mean. Error bars represent ± SE.

**FIGURE 4:**
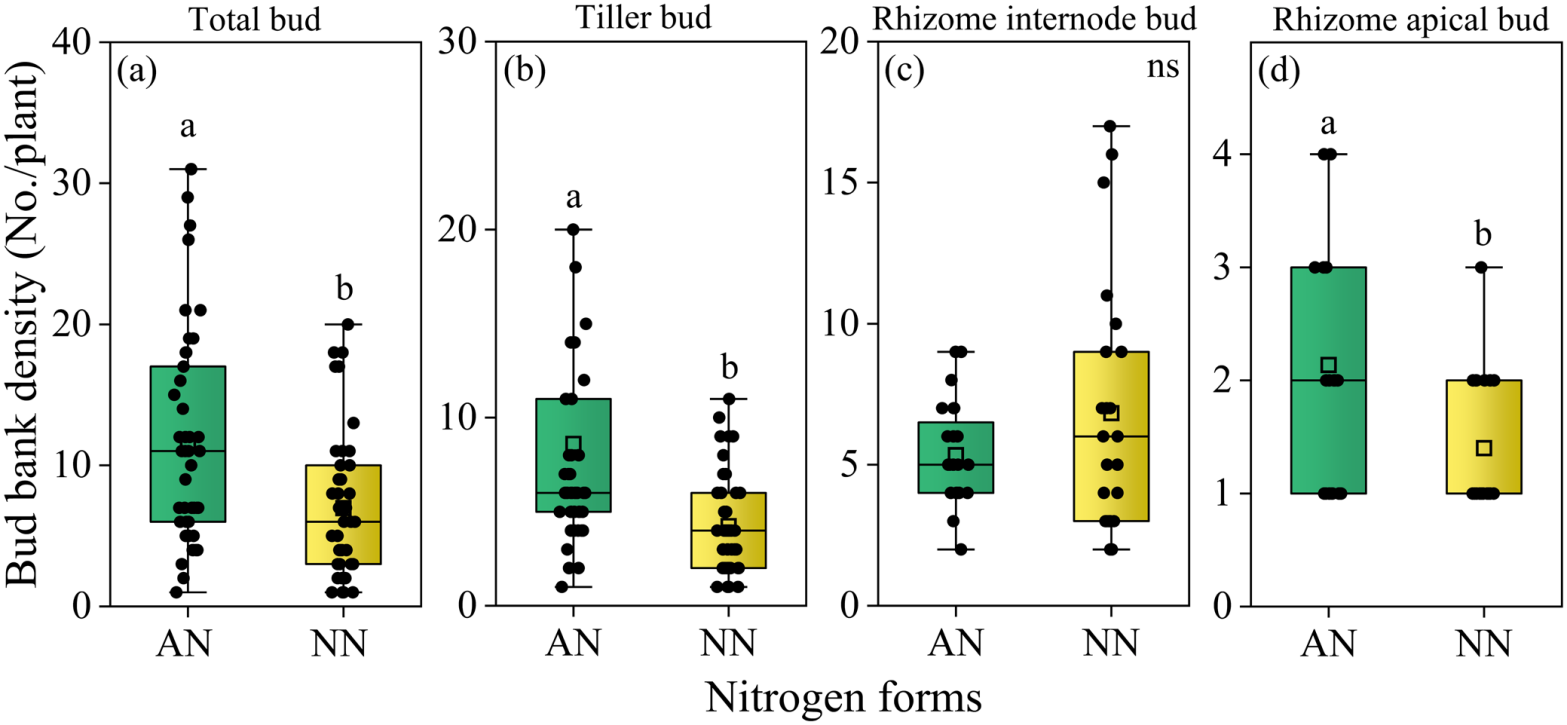
Variation in bud bank density under different nitrogen form treatments. Different lowercase letters indicate significant (*p* < 0.05) differences between different nitrogen forms; ns: Not significant (*p* > 0.05); AN and NN stand for ammonium nitrogen and nitrate nitrogen, respectively. The box shows the interquartile range (IQR) with the median line; whiskers extend to the most extreme data points within 1.5 × IQR of the box. The square symbol within the box indicates the mean. Error bars represent ± SE.

Nitrogen level significantly influenced the densities of total bud and tiller bud of *G. spiculosa* (Table 2, *p* < 0.001), which increased with increasing nitrogen levels (Figure 3a,b). Nitrogen form also significantly affected the total bud, tiller bud bank, and rhizome apical bud (Table 2, *p* < 0.05). Specifically, compared to nitrate nitrogen treatments, ammonium nitrogen treatments significantly increased the densities of the total bud, tiller bud, and rhizome apical bud (Figure 4, *p* < 0.05). Flooding period had no significant impact on the density of the bud bank (Table 2 and Figure S3, *p* > 0.05).

**TABLE 2:** Results of analysis of covariance (ANCOVA) of initial plant height (H, covariate), nitrogen level (NL), nitrogen form (NF), flooding period (FP), and their interactions on bud bank density.

	Total buds			Tiller buds			Rhizome internode buds			Rhizome apical buds		
	df	*F*	*p*	df	*F*	*p*	df	*F*	*p*	df	*F*	*p*
H (covariate)	1	0.143	0.707	1	3.702	0.059	1	0.431	0.517	1	0.405	0.531
NL	3	12.900	**< 0.001**	3	10.539	**< 0.001**	3	1.343	0.281	3	2.173	0.120
NF	1	15.728	**< 0.001**	1	27.525	**< 0.001**	1	2.349	0.137	1	5.302	**0.031**
FP	2	1.312	0.277	2	2.503	0.090	2	0.821	0.451	2	1.942	0.167
NL × NF	3	0.931	0.431	3	5.109	**0.003**	2	2.909	0.072	3	1.886	0.161
NL × FP	6	0.663	0.680	6	2.174	0.056	5	1.174	0.348	4	1.993	0.131
NF × FP	2	2.254	0.114	2	2.336	0.105	2	0.579	0.567	2	0.915	0.415
NL × NF × FP	5	1.615	0.169	6	1.063	0.394	3	0.421	0.740	3	0.574	0.638

*Note:* The bolded value represents *p* < 0.05.

The interaction between nitrogen level and nitrogen form exhibited a significant effect only on the tiller bud density (Table 2, *p* < 0.01). The higher the nitrogen level, the greater the difference in the effect of two nitrogen forms on tiller bud density (Figure S4). The interaction effect of flooding period and nitrogen treatment on bud bank density was not significant (Table 2, *p* > 0.05).

### 3.3 Environmental Explanation of Bud Banks Variation

In natural habitats, the vegetation zones were pulled together and only the effects of soil and water properties were considered. It was found that neither total bud density, tiller bud density, rhizome internode bud density, nor rhizome apical bud density showed significant responses to soil nutrients, water nutrients, or water depth (Figure S5).

**FIGURE 5:**
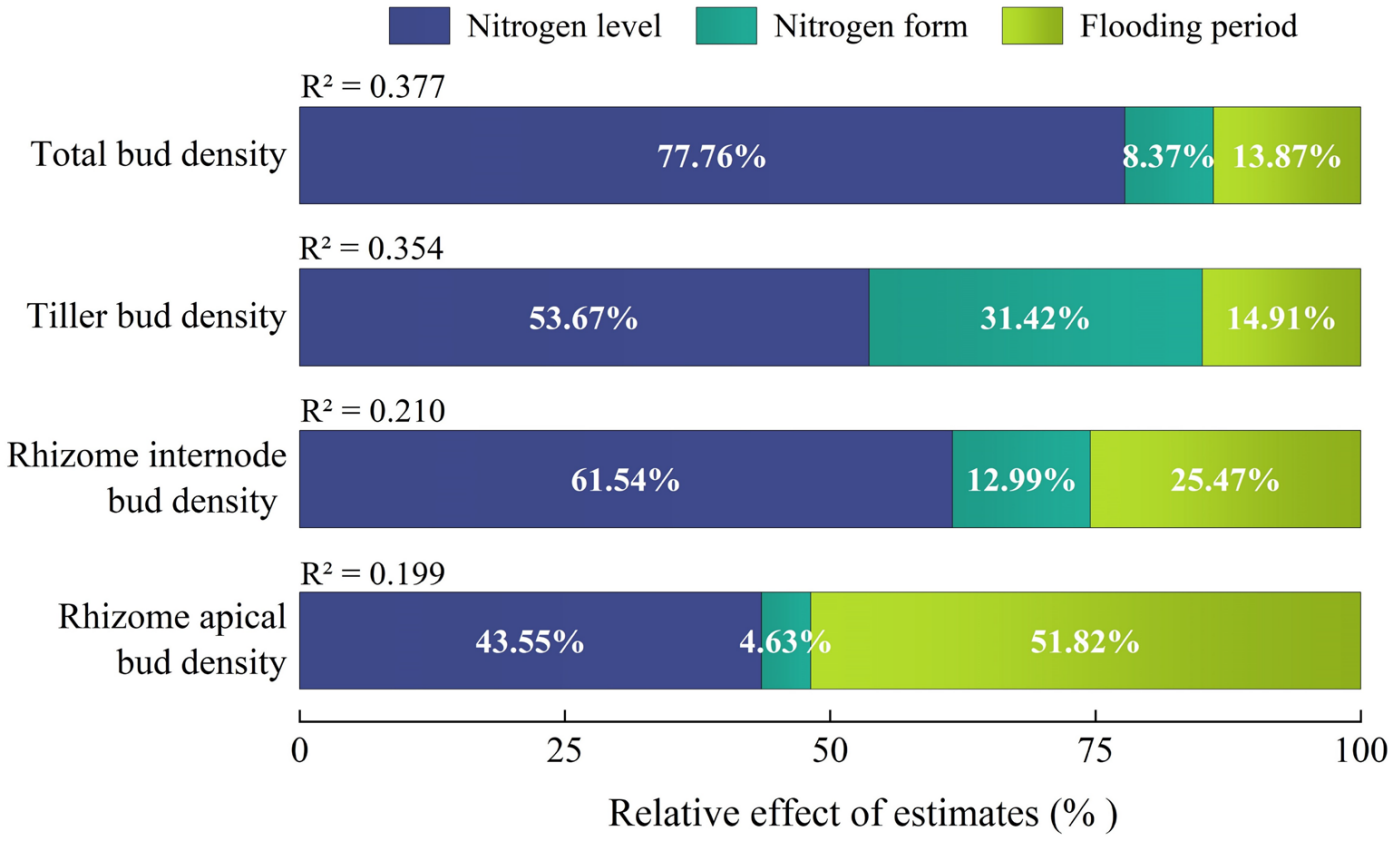
Relative effect of nitrogen level, nitrogen form, and flooding period on bud bank density.

In pot experiments, flooding and nitrogen treatments accounted for 37.7%, 35.4%, 21.0%, and 19.9% of the total variation in total bud, tiller bud, rhizome internode bud, and rhizome apical bud densities, respectively. Specifically, nitrogen level was identified as the most important factor affecting the densities of total bud, tiller bud, and rhizome internode bud, with relative effects of 77.76%, 53.67%, and 61.54%, respectively. Flooding period exerted the most significant influence on rhizome apical bud density, with a relative effect of 51.82%, and was also the second most critical factor affecting total bud and rhizome internode bud densities, with relative effects of 13.87% and 25.47%, respectively. Nitrogen form was the second most influential factor of tiller bud density variation (with relative effect of 31.42%), while it had a small impact on total bud, rhizome internode bud, and rhizome apical bud, with relative effects of 8.37%, 12.99%, and 4.63%, respectively (Figure 5).

## 4 Discussion

### 4.1 Bud Bank variation in Natural Vegetation Zones

In natural habitats, the bud bank of *G. spiculosa* was dominated by rhizome internode buds, and the bud density decreased first and then increased along the vegetation zone from wet meadow to tussock marsh and then to marsh. This trend suggests that *G. spiculosa* tends towards a guerrilla growth strategy in wild habitats (especially in marsh zones), which involves rapidly occupying living space through rhizome expansion and the sprouting of rhizome internode buds (Zuo et al. 2023). However, Deng et al. (2013) found that *Carex brevicuspis* in Dongting Lake wetland primarily employs a “phalanx” strategy to adapt to habitats with high water levels by observing its bud bank. This discrepancy may be attributed to the loose grass root layer in Sanjiang Plain marsh. The gaps among roots create advantageous conditions for the rapid growth and lateral expansion of rhizomes, which in turn enhance rhizome internode bud production and lead to a larger bud bank. Additionally, the marsh zones with relatively low elevation frequently suffer from flooding stress caused by snowmelt water and seasonal precipitation; a larger bud bank serves as a buffer and assists plants in completing population renewal when the stress is alleviated (Wu et al. 2020). Wet meadows characterized by low water levels and hard soil substrate may potentially limit rhizome growth due to increased roughness (Klimešová et al. 2023) and result in a decrease in bud bank density. Tussock marsh tends to foster species with high‐density root systems such as *C. meyeriana* , and these species often form elevated mounds consisting of root necromass (Curasi et al. 2023). Our field observations revealed that these mounds exhibit low porosity, which inhibits the growth of *G. spiculosa* rhizomes.

### 4.2 Relationship Between Bud Bank Dynamics and Environmental Factors

Our results showed that the bud density of *G. spiculosa* experienced minor influences from environmental factors in natural habitats. This is consistent with the findings in a meta‐analysis study of bud bank density in response to environmental perturbations (Fang et al. 2024). However, this might also be affected by the limited size of our sample. We observed that water depth serves as a crucial factor influencing bud density in the field and exhibits a negative correlation. Although this relationship is not proved in our data, this observation suggests that the species tends to prioritize the allocation of limited resources to rhizome and rhizome bud formation at high water levels. Through lateral expansion via developed rhizomes, *G. spiculosa* can rapidly occupy favorable spaces (Ievinsh 2023; Bai et al. 2009), thereby establishing its status as a dominant species in marsh ecosystems.

### 4.3 Bud Bank variation under Flooding Periods and Nitrogen Addition treatment

In the pot experiment, the density of tiller buds was higher than that of rhizome internode buds, indicating that *G. spiculosa* utilized a phalanx growth strategy. This strategy facilitates the plant to optimize resource utilization within confined living spaces (Ye et al. 2006) or to proliferate rapidly and evade unfavorable patches where resources are scarce or pressures are high (Wang et al. 2017). The flooding period exhibited no significant impact on the bud banks of *G. spiculosa*, and this can be attributed to the robust flood tolerance of this species, which can increase adventitious roots and enhance the absorption and utilization efficiency of oxygen in the roots under flooding conditions to mitigate the stress (Ayi et al. 2016).

As the nitrogen level increased, the total bud and tiller bud density of *G. spiculosa* exhibited a corresponding increase. This finding aligns with previous research on the effects of nitrogen fertilizer on the bud banks of *Leymus chinensis* (Yu et al. 2022). As one of the essential macronutrients for plant growth, nitrogen may be an inducing factor for bud dormancy or germination (Tomlinson and O'Connor 2004). Sufficient nitrogen supply provides a stable growth environment, thereby effectively promoting the formation and expansion of bud banks. Additionally, our study reveals that tiller buds respond more sensitively to nitrogen levels compared to rhizome buds, which concurs with the findings on *L. chinensis* (Yu et al. 2022). This observation further underscores that plants adopt phalanx growth strategies in resource‐rich environments to optimize the utilization of environmental resources.

Compared to nitrate nitrogen, ammonium nitrogen significantly enhanced the total bud, tiller bud, and rhizome apical bud density of *G. spiculosa*. This result stems from the differences in the mechanisms of absorption and utilization of ammonium and nitrate nitrogen by plants. Ammonium nitrogen, as a cation, is directly assimilated into amino acids or other nitrogen‐containing organic compounds within root cells and can be more readily absorbed by plant roots. In contrast, nitrate nitrogen necessitates a series of complex reduction processes to be utilized by plants and demands greater energy expenditure (Subbarao and Searchinger 2021). Furthermore, cell division may be faster under ammonium nitrogen than under nitrate nitrogen (Bloom et al. 2003). Fu et al. (2021) found that ammonium nitrogen treatment significantly elevated the tiller number of rice compared to nitrate nitrogen treatment. So, *G. spiculosa* should also be a plant preferring ammonium nitrogen. The interaction between nitrogen forms and nitrogen levels only significantly affects the density of tiller buds. Specifically, as the nitrogen level rises, the promotional effect caused by ammonium nitrogen treatment is further augmented and this may be related to the regulation of endogenous hormone synthesis and signal transduction et al.

### 4.4 Nitrogen Conditions Rather Than Flooding Determine Bud Bank Density

In comparison to flooding, the addition of nitrogen exerts a more significant influence on bud banks. This is in alignment with the result of Li et al. (2021) that the augmentation of available nitrogen in the habitat, as opposed to an elevation in soil water content, was the primary factor accounting for variations in asexual reproduction. On the one hand, this may be attributed to the bud bank's robust resistance and resilience against water level variations (Fang et al. 2024). Lou et al. (2018) have proved that this species has a wide water depth amplitude. On the other hand, this may also be related to the flooding and nitrogen conditions set in our experiment. The relative effects of water and nitrogen on the bud bank will continue to be studied in the future by setting other different factor conditions. Even in the current results, the flooding period is still an environmental factor that cannot be ignored because of its impact on the rhizome apical bud density of *G. spiculosa* (Figure 5), indicating that these buds are highly sensitive to flooding period variations, with peak growth period flooding being particularly conducive to their formation.

## 5 Conclusion

In this study, we demonstrated that *G. spiculosa* dynamically adjusts bud bank composition and density as a central strategy to cope with habitat conditions variations. In natural wetlands, rhizome internode buds accounted for 91% of total density, and bud density in marsh was higher than that in tussock marshes and wet meadows. In pot experiments, the proportion of tiller buds exceeds that of rhizome internode buds. The densities of total buds and tiller buds increased with increasing nitrogen levels, and the effect of ammonium nitrogen is higher than that of nitrate nitrogen. Flooding periods selectively promoted rhizome apical buds. Overall, the impact of nitrogen is greater than that of flooding. These findings suggest that nitrogen fertilizers (including nitrogen form and level) and flooding periods should be attached with more importance in wetland restoration to maintain bud bank–mediated regeneration capacity of plant populations. Looking forward, future research should unravel the physiological thresholds of bud dormancy and activation under nitrogen‐water synergies, which will enhance our comprehension of plant adaptation strategies based on bud bank regulation mechanisms.

## Author Contributions

**Mengdie Zhou:** data curation (equal), formal analysis (lead), investigation (equal), methodology (lead), visualization (lead), writing – original draft (lead). **Changwei Zhang:** data curation (equal), investigation (equal). **Xianglong Jin:** data curation (supporting), investigation (supporting). **Qiyao Zhang:** data curation (supporting), investigation (supporting). **Peng Zhang:** data curation (supporting), investigation (supporting). **Anni Bao:** formal analysis (supporting), visualization (supporting). **Yanjing Lou:** conceptualization (lead), funding acquisition (lead), supervision (lead), writing – review and editing (lead).

## Conflicts of Interest

The authors declare no conflicts of interest.

## Supporting information


Data S1.

Data S2.

## Data Availability

The datasets for this study are accessible in the Supporting Information—S1.
