# Supplementary material for: Response of Bud Banks of a Freshwater Herbaceous Marsh Plant (Glyceria Spiculosa) to Heterogeneous Habitat: Field Observations and Experiment
Source: Ecol Evol. 2025 Jun 17;15(6):e71522. doi: 10.1002/ece3.71522 (PMC12173836; doi:10.1002/ece3.71522)
Supplement: Supplementary file 1 — Data S1. [file ECE3-15-e71522-s001.docx]

**Supplementary Material Legends**

Fig.S1 Study area

Fig.S2 The setups for the pot experiment.

Fig. S3 Variation in bud bank density under different flooding period treatments.

Fig. S4Variation in tiller bud density under the interaction of nitrogen form and nitrogen level.

Fig. S5 Correlation analysis between environmental factors and bud bank densities.

Table S1 Absolute amounts of (NH₄)₂SO₄ and Ca(NO₃)₂·4H₂O for different nitrogen levels.

Table S2 Composition of modified Hoagland’s nutrient solution.


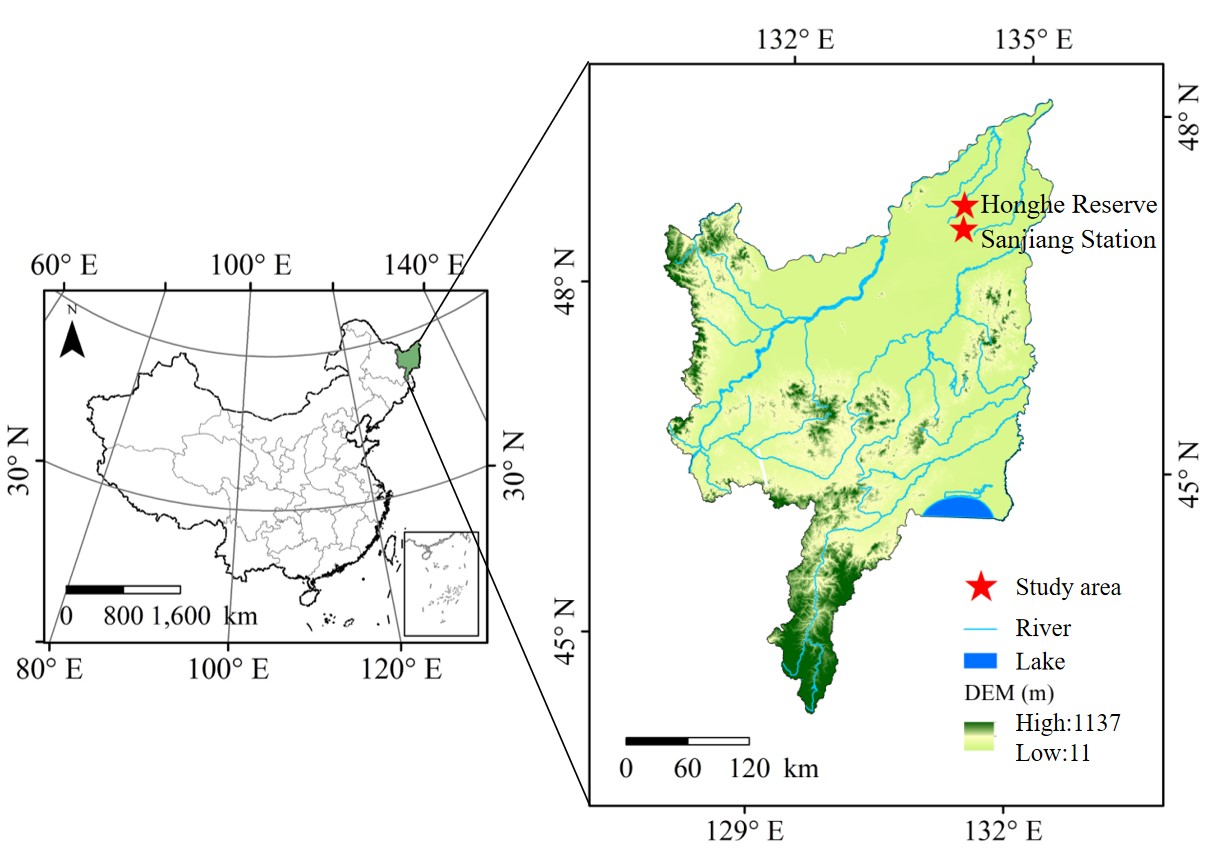


Fig. S1 Study area


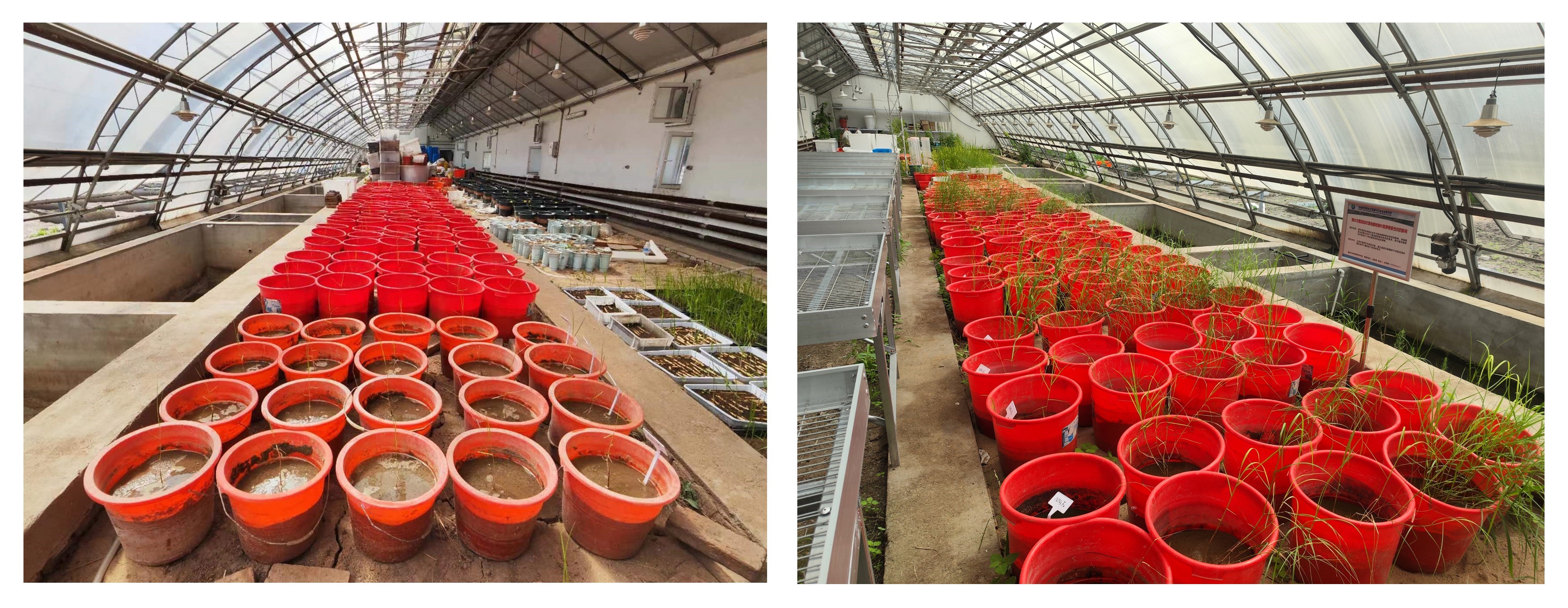


Fig. S2 The setups for the pot experiment.


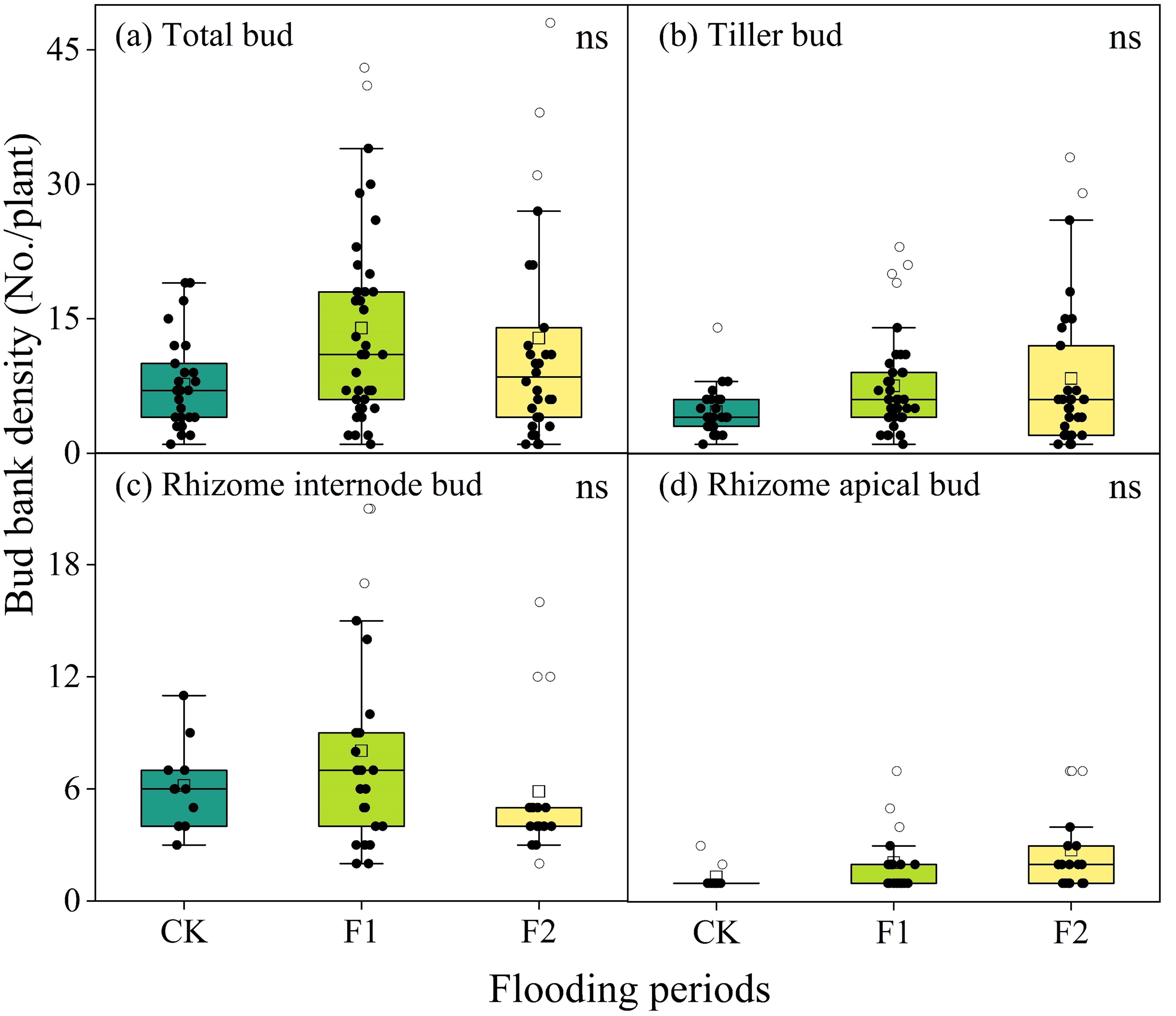


Fig. S3 Variation in bud bank density under different flooding period treatments. ns: not significant (*p* > 0.05); CK, F1 and F2 represent no flooding throughout, early flooding (0-5 weeks flooding) and late flooding (6-10 weeks flooding), respectively.


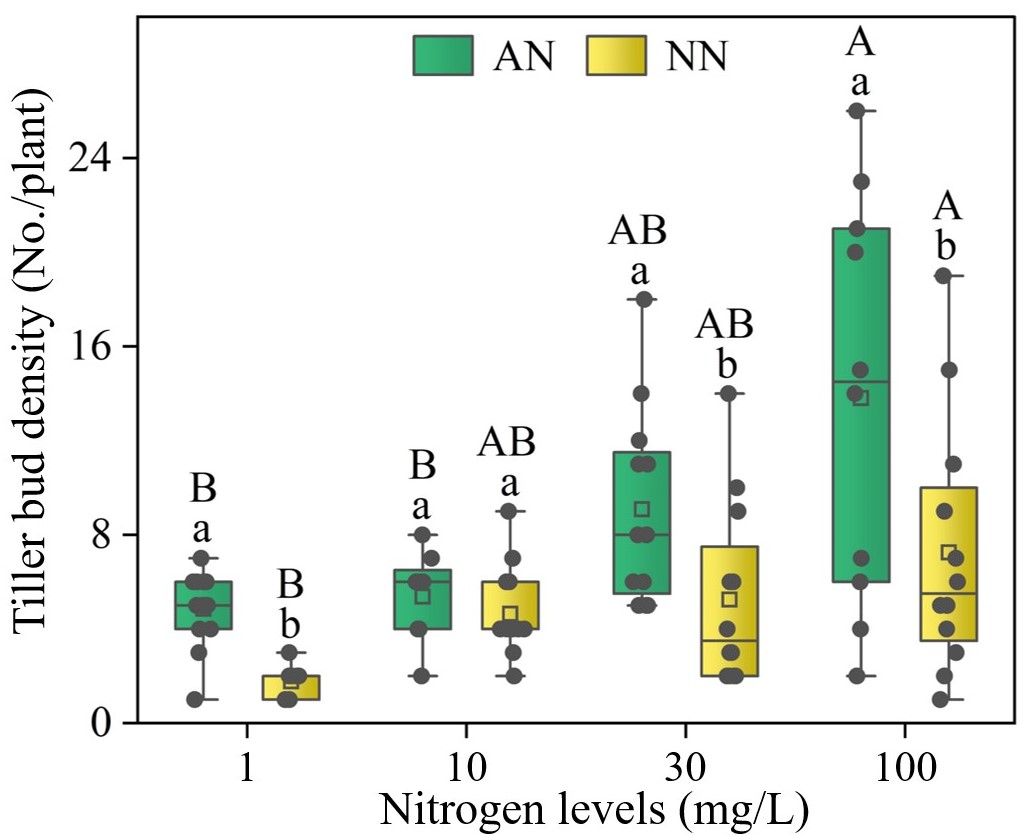


Fig. S4 Variation in tiller bud density under the interaction of nitrogen form and nitrogen level. Different uppercase and lowercase letters indicate significant differences (*p* < 0.05) between different nitrogen levels and nitrogen forms, respectively; AN and NN stand for ammonium nitrogen and nitrate nitrogen, respectively.


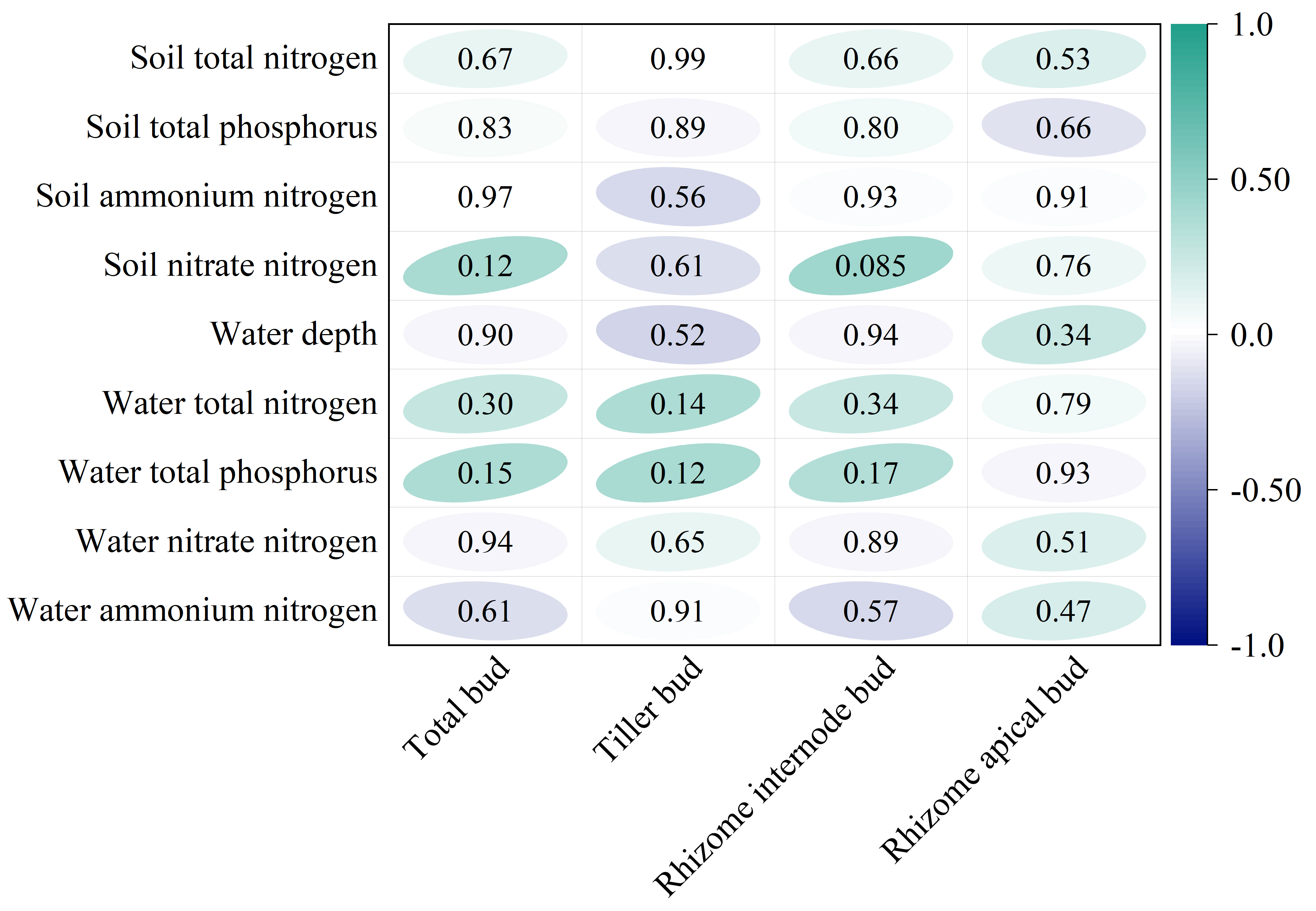


Fig. S5 Correlation analysis between environmental factors and bud bank densities. Numbers within ellipses denote *p*-values. Green hues indicate positive Pearson’s correlation coefficients (*r* > 0), while blue hues represent negative correlations (*r* < 0). Color intensity corresponds to the absolute value of *r*, with darker shades indicating stronger correlations

Table S1 Absolute amounts of (NH₄)₂SO₄ and Ca(NO₃)₂·4H₂O for different nitrogen levels.

| Nitrogen level (mg/L) | (NH₄)₂SO₄ (mg/L) | Ca(NO₃)₂·4H₂O (mg/L) |
| --- | --- | --- |
| 1 | 4.72 | 8.43 |
| 10 | 47.15 | 84.32 |
| 30 | 141.45 | 252.96 |
| 40 | 471.50 | 843.20 |

Table S2 Composition of modified Hoagland’s nutrient solution.

| **Substance** | **Concentration（μM / L）** |
| --- | --- |
| KH_2_PO_4_ | 500.000 |
| KCl | 3000.000 |
| CaCl_2_ | 2000.000 |
| MgSO_4_ 7H_2_O | 1000.000 |
| H_3_BO_3_ | 23.130 |
| MnCl_2_ 4H_2_O | 4.570 |
| ZnSO_4_∙7H_2_O | 0.382 |
| CuSO_4_∙5H_2_O | 0.160 |
| MoO_3_ | 0.070 |
| Fe-EDTA | 9.000 |
